# Superior Effects of Modified Chen-Style Tai Chi versus 24-Style Tai Chi on Cognitive Function, Fitness, and Balance Performance in Adults over 55

**DOI:** 10.3390/brainsci9050102

**Published:** 2019-05-04

**Authors:** Liye Zou, Paul D. Loprinzi, Jane Jie Yu, Lin Yang, Chunxiao Li, Albert S. Yeung, Zhaowei Kong, Shin-Yi Chiou, Tao Xiao

**Affiliations:** 1 Lifestyle (Mind-Body Movement) Research Center, College of Sports Science, Shenzhen University, Shenzhen 518060, China; 2 Department of Health, Exercise Science and Recreation Management, The University of Mississippi, University, MS 38677, USA; pdloprin@olemiss.edu; 3 Department of Sports Science and Physical Education, The Chinese University of Hong Kong, Shatin, Hong Kong, China; jieyu0203@gmail.com; 4 Cancer Epidemiology and Prevention Research, Alberta Health Services, Calgary, AB T2S 3C3, Canada; lin.yang@ahs.ca; 5 Departments of Oncology and Community Health Sciences, Cumming School of Medicine, University of Calgary, Calgary, AB T2N 4Z6, Canada; 6 Physical Education and Sport Science Academic Group, National Institute of Education, Nanyang Technological University, 1 Nanyang Walk, Singapore 637616, Singapore; cxlilee@gmail.com; 7 Depression Clinical and Research Program at the Massachusetts General Hospital, Harvard Medical School, Boston, MA 02115, USA; ayeung@mgh.harvard.edu; 8 Faculty of Education, University of Macau, Macao, China; zwkong@um.edu.mo; 9 School of Sport, Exercise and Rehabilitation Sciences, University of Birmingham, Birmingham B15 2TT, UK; s.chiou12@imperial.ac.uk; 10 College of Mathematics and Statistics, Shenzhen University, Shenzhen 518060, China

**Keywords:** mind-body exercise, aging, cognition, balance, Tai Chi

## Abstract

Background: Cognitive decline and balance impairment are prevalent in the aging population. Previous studies investigated the beneficial effects of 24-style Tai Chi (TC-24) on either cognitive function or balance performance of older adults. It still remains largely unknown whether modified Chen-style TC (MTC) that includes 18 complex movements is more beneficial for these age-related health outcomes, as compared to TC-24. Objective: We investigated if MTC would show greater effects than TC-24 on global cognitive function and balance-related outcomes among older adults. Methods: We conducted a randomized trial where 80 eligible adults aged over 55 were allocated into two different styles of Tai Chi (TC) arms (sixty-minute session × three times per week, 12 weeks). Outcome assessments were performed at three time periods (baseline, Week 6, and Week 12) and included the Chinese Version of the Montreal Cognitive Assessment (MoCA) for overall cognitive function, One-leg Standing Test (LST) for static balance, Timed Up and Go Test (TUGT) for dynamic balance, chair Stand Test (CST) for leg power, and the six-meter Walk Test (6MWT) for aerobic exercise capacity. Results: Compared to TC-24 arm, MTC arm demonstrated significantly greater improvements in MoCA, LST, TUGT, CST, and 6MWT (all *p* < 0.05). Conclusions: Both forms of TC were effective in enhancing global cognitive function, balance, and fitness. Furthermore, MTC was more effective than TC-24 in enhancing these health-related parameters in an aging population.

## 1. Introduction

The aging population is rising in China and the number of people aged ≥ 55 years account for more than 21% of the total population [1]. With increasing longevity, it is estimated that almost half of the aging population will suffer at least one physical or mental illness due to normal aging [2]. An age-related illness that is highly prevalent in the aging population is cognitive deterioration [3]. Brain tissue atrophy is associated with reduced cognitive function [4,5]. This progressive cognitive decline and impairment could lead to increased risk of developing dementia, especially Alzheimer’s disease, which will produce significant economic and societal burden [6]. To slow down and prevent the progression of age-related cognitive decline, early intervention programs should, for example, include activities that minimize cardiovascular risk factors, employ cognitively stimulating activities, and employ exercise activities in a social environment [7,8,9]. 

Tai Chi (TC) is a typical form of traditional Chinese health-promoting exercise [10,11,12], with more than 3000 years of history [13,14,15]. Despite its complicated movement sequences, TC involves mild to moderate exercise intensity and is suitable for aging people with low exercise tolerance [16,17,18]. TC involves both social interaction and cognitive stimulation, embodied within the instruction and movement characteristics, respectively [19,20]. Teaching TC involves a bi-directional communication process in which both students and instructor are typically involved with cognitive and emotional investment for maximizing the learning outcome [21,22,23]. In addition, social–psychological interactions are emphasized to enhance cognitive stimulation and movement skill proficiency [23]. Notably, however, very few TC studies have investigated the psycho–social effects of TC [24]. To fill this gap, Mortimer and colleagues conducted a well-designed controlled trial in which 120 healthy Chinese older adults were randomized into one of four groups (TC, walking, social interaction, and no-intervention) for a forty-week intervention period [25]. Both the TC and social interaction groups demonstrated significant improvements in brain volume and cognitive behavioral outcomes, whereas these positive results were not observed in the walking and control groups [25]. These authors suggested that social interaction through TC instruction may play an essential role in facilitating cognitive performance [25]. 

Previous studies have investigated the effects of TC and brisk walking on metabolic expenditure and cardiovascular (CV) health [26,27]. Although TC induces 46% less metabolic cost than brisk walking for one bout of 10 minutes, they produced similar benefits on reducing cardiovascular risk factors over 12 weeks of exercise (e.g., blood cholesterol, body composition, VO2 max) [26,27]. Apart from these CV health benefits, adequate cognitive function is necessary to stabilize the center of gravity during TC performance (e.g., dynamic weight shifting and single limb support) [28]. In order to facilitate adequate psychological attention, mindfulness and diaphragmatic breathing during TC training should be coordinated [29,30]. Further, refined attentional skills are associated with emotion-regulation processes involving the executive control network [31,32,33]. These potential therapeutic elements inherent in TC may provide additional cognitive stimulation. Taken together, it is reasonable to suppose that the critical elements inherent in TC seems to exert beneficial effects on cognitive function and/or protect against age-related cognitive decline. 

Cognitive decline is highly prevalent in older adults [34,35,36], and aging is generally accompanied by fall-related incidents (injuries and mortality) as well [37,38]. Unfortunately, cognitive decline and degenerating balance among the aging population were generally accepted as separate problems in the earlier literature [39], and thus, were treated independently. Furthermore, previous TC studies focused on either the protective effects of TC on cognitive function or balance function. However, a growing number of studies support postural control and cognition as highly interdependent issues [40,41]. Balance and gait pattern are no longer considered automated motor action, but rather are activities that require higher-order cognition [42]. Thus, we included both cognition and balance outcome measures in the same study. Notably, the majority of previous studies on this topic used 24-style TC (TC-24) as the exercise intervention program. It largely remains unknown if other styles of TC form are more beneficial for cognitive function and balance as compared to TC-24. For example, Chen-style TC involves more complex choreography with skipping moves, which may provide more cognitive and balance challenges, potentially having a greater effect on cognitive function and balance. Thus, to address this gap in the literature, a randomized controlled trial was conducted to investigate the effects of TC-24 versus modified Chen-style TC (MTC) on cognitive function and balance performance among older adults. 

## 2. Methods 

**Figure 1:**
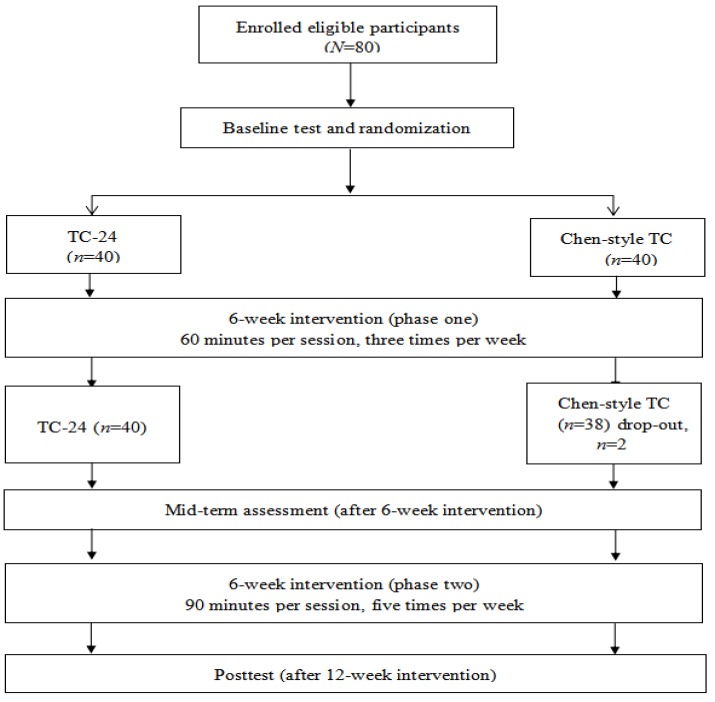
Flowchart of the study. TC = Tai Chi; TC-24 = 24-style Tai Chi.

This study was a twelve-week randomized trial with two different styles of TC practice, including TC-24 and MTC as experimental conditions. All outcome variables were assessed at baseline, mid-term of intervention (6 weeks), and immediately after the intervention (12 weeks) (see Figure 1). The study was approved by the ethics committee of the university and all participants provided consent prior to participation. 

### 2.1. Study Participants

Eighty Chinese adults (age range: 55–79 years old) were recruited and met the following criteria: (1) were male or female aged at 55 years or above; (2) were healthy and able to participate in exercise; (3) had normal cognitive function, as indicated by the Mini Mental State Examination score of ≥ 26; and (4) had no regular practice of TC or other exercise previously. Participants who had serious diseases (e.g., cardiovascular disease, dementia, clinical depression, and/or sequela of apoplexy) or a history of drug or alcohol abuse were excluded. Based on a Random Integer Generator, the participants were allocated randomly into two experimental conditions (either TC-24 or MTC group) matched by gender, with 40 participants (20 males, 20 females) in each arm. No allocation concealment occurred in the present study. Participants were asked to maintain their usual routine (e.g., diet, activities of daily living) throughout the study. Given that two participants in the MTC arm dropped out from the intervention, data analyses were processed using data from the remaining 78 participants. 

### 2.2. Intervention

The duration of intervention was a total of 12 weeks. Participants in the two experimental arms (TC-24 vs. MTC) were instructed to practice one specific style of TC (24-style vs. MTC). Simplified TC consists of 24 postures and takes about six minutes to perform. Eighteen Postures [43] were selected from Chen-style 56-form TC and choreographed by an experienced TC instructor. Detailed information about the selected postures of MTC are reported in our previous study [44]. The intervention consisted of two phases, with frequency and duration of practice being increased progressively. In the first phase (from week 1 to week 6), each session of practice lasted 60 minutes, including 10 minutes of warm-up, 40 minutes of TC practice (including learning of new content), and 10 minutes of cool-down, three times per week for six weeks. In the second phase (from week 7 to week 12), each session of practice lasted for 90 minutes, including 10 minutes of warm-up, 70 minutes of TC practice, and 10 minutes of cool-down, five sessions (rest on Wednesday and Saturday) per week for six weeks. The intervention in the two arms was led by the same TC instructor who had more than 15 years of TC teaching experience. The instructor explained and demonstrated the movements required in different experimental conditions and the participants followed and practiced independently afterwards.

### 2.3. Outcome Measures

#### 2.3.1. Demographic Information

Demographic information included date of birth, sex, and educational level, which were reported by the participants. Body height and weight (Healthometer 402KL Beam Scale weight/Height Rod) were objectively assessed at baseline by trained research assistants.

#### 2.3.2. Cognitive Function

General cognitive ability was assessed using the Chinese version of the Montreal Cognitive Assessment (MoCA) [45], which is a 30-item test to evaluate various components of cognition (i.e., executive function, language, orientation, memory, and abstraction) and has been widely used to assess cognitive impairments in adults [46]. It has good criterion-related validity (Pearson Correlation Coefficient between MoCA and Mini-mental state examination, *r* = 0.787) and reliable internal consistency (Cronbach alpha = 0.807). The total score of MoCA ranges from 0 to 30, with a higher score representing better cognitive ability.

#### 2.3.3. Balance

One-leg standing test was used to assess static balance. The participants were asked to close their eyes, stand on their preferred leg, lift the knee of the other leg to approximately 90°, keep their arms by their sides, and maintain balance without using any assistive device. The test was over when the stance foot shifted or when the lifted foot was replaced on the ground, whichever occurred first. Each participant had three attempts for each leg. The duration of standing (in seconds) was recorded in each attempt and the best (longest) score was chosen for analysis [47].

Timed up and go test (TUGT) was used to assess dynamic balance. It measures the time, in seconds (s), taken by a participant to stand up from a standard arm chair, walk three meters, turn around, walk back to the chair, and sit down without physical assistance. The participants were instructed to be seated on the chair (approximate seat height of 45 cm) with their arms resting on the chair’s arms, and to stand up on the “go” command. The stopwatch was started on the “go” command and stopped as the participant sat down [48]. Each participant had three attempts, with the best attempt being recorded for data analyses.

#### 2.3.4. Functional Fitness of Lower Limbs

Chair Stand Test (CST) was used to measure the strength and endurance of the lower limbs. The participants were seated on a standard armless chair (approximate seat height of 45 cm; placed against a wall) with their back in an upright position and their feet flat on the floor. With their arms folded across the chest, they were asked to stand completely up and then completely back down as fast as possible after the “1, 2, 3, go” command. The outcome measure was the time (in seconds) they spent in performing five complete chair stands continuously. All subjects had three attempts with a short break (approximate 3 minutes) in between. The best (fastest) score was selected for analysis [49]. All participants performed this test using the same chair and with similar ambient conditions. 

#### 2.3.5. Aerobic Exercise Capacity

Aerobic exercise capacity was evaluated by the six-meter walk test (6MWT). A smooth and flat twelve-meter walkway was marked out on the floor with tape markers being placed at the 0, 3, 9, and 12 m points along the walkway. Standing at the zero-meter point, subjects were asked to walk to the end of the walkway (marked at 12 m point) at their own comfortable speed without stopping. A tester timed the participants’ walking (in seconds) over the 6 meters (from 3 to 9 m point) using a stopwatch [50]. The first and last three meters of the walk were not timed in consideration of changes in velocity that occur when people start and stop walking. All participants had two attempts with the best (fastest) score being used in data analyses. 

### 2.4. Statistical Analysis

**Figure 2:**
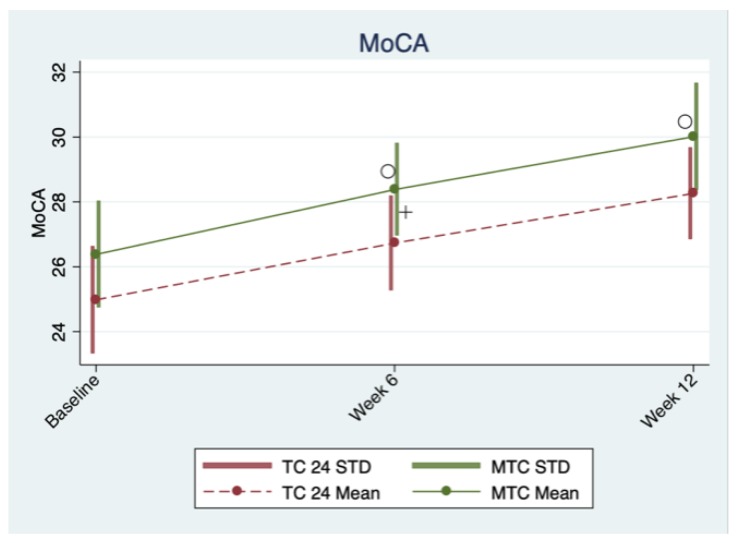
Interaction plots for indices of cognitive function.

**Figure 3:**
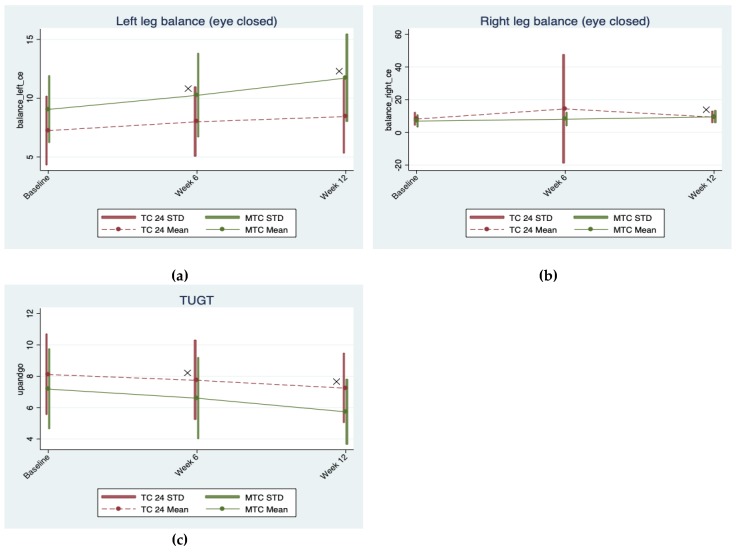
Interaction plots for indices ((**a**) left leg balance, (**b**) right leg balance, and (**c**) TUGT) of balance.

**Figure 4:**
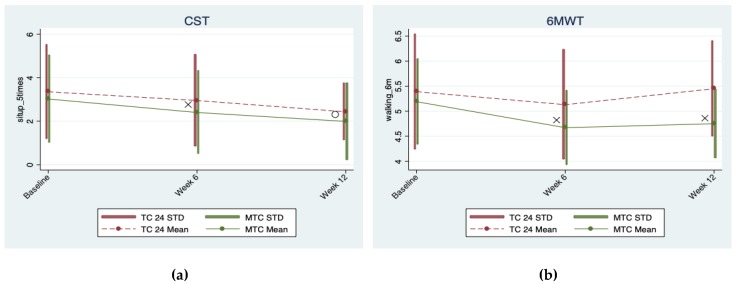
Interaction plots for indices ((**a**) CST and (**b**) 6MWT)) of physical fitness.

The data were analyzed using Stata [51]. For each outcome variable, a population average model using generalized estimating equations, or GEE [52], was built to assess the intervention effects of the two Tai-Chi styles over time, while adjusting for confounders. Correlations among the three repeated measures of each outcome variable taken within each participant were assumed to be exchangeable, and parameter estimates and their robust standard errors were obtained with the Stata xtgee command for the GEE approach, for which the distribution assumption of the dependent variable is not required [53]. Main and interaction effects terms for both the repeated measure time factor and the Tai-Chi style group factor were first included in the GEE model for each outcome, and then we assessed the significance of the time-arm interaction for each outcome. If the time-arm interaction effects for an outcome were significant, we reported estimated coefficients and their *p*-values for both the main effects and the interaction effects for this outcome; otherwise if the time-arm interaction effects of a model for an outcome were not significant, a reduced model with only the main effects terms was re-fit for this outcome (i.e., removing the time-arm interaction from the GEE model), and we reported estimated coefficients and their p-values for the main effects from the reduced model for this outcome. Two baseline variables (i.e., Body Mass Index [BMI] and Education Level) in our sample were significantly different between the two Tai-Chi arms, and were thus included as main effect terms in all GEE models (with or without time-group interactions) to control for their confounding effects. Statistical significance was set at a nominal alpha of 0.05. The interaction plots of the outcome variables are given in Figure 2, Figure 3 and Figure 4. To exclude the confounding effects in these Figure illustrations, we generated these Figures based on outcome values adjusted for the effects of the two confounders (i.e., BMI and Education Level); the adjusted outcome values were obtained by subtracting the estimated effects of these two confounders from the outcome values. The means and standard deviation ranges of these adjusted outcomes at different points for the two Tai Chi groups are illustrated in these Figures, along with different symbols to indicate significance of different types of effects (symbol “o” stands for time effect; symbol “+” stands for group effect; symbol “×” stands for time × group interaction effect).

## 3. Results

We only found significant differences between two TC arms on educational level and BMI (*p* < 0.05). Thus, the two variables were included as main effect terms in all GEE models to adjust for their confounding effects. Detailed demographic information is presented in Table 1. 

### 3.1. Cognitive Function

There was no significant time × arm interaction effect, and hence only the main effects of time and arm were included in the model. Overall, participants in two Tai-Chi style arms exhibited increases in MoCA both at 6 weeks (1.872, 95% CI 1.641 to 2.101, *p* < 0.0005) and at 12 weeks (3.449, 95% CI 3.152 to 3.745, *p* < 0.0005) from baseline. Compared to the TC-24 arm, mean MoCA scores were constantly higher (0.487, 95% CI 0.044 to 0.930, *p* = 0.031) in the MTC arm throughout the study period (Table 2 and Table 3 visually illustrated in Figure 2). Detailed information about study results are presented in Table 2 and Table 3. 

### 3.2. Balance Performance

Three indices for balance (left/right leg balance and TUGT) displayed increases through the study period, except for right leg balance at the six-week assessment (Table 2). Significant time × arm interaction effects were detected at both 6 weeks and 12 weeks, such that the increase in left leg balance in the MTC arm was 0.446 (95% CI 0.012 to 0.879, *p* = 0.044) higher from baseline to 6 weeks and 1.485 (95% CI 0.938 to 2.033, *p* < 0.0005) higher from baseline to 12 weeks compared to that in the TC-24 arm. Similar time × arm interaction was observed in right leg balance, but only at 12 weeks. Compared to TC-24 arm, the increase in right leg balance was 1.385 (95% CI 0.723 to 2.046, *p* < 0.0005) higher from baseline to 12 weeks in the MTC arm. The time × arm interaction for TUGT was significant at both 6 weeks and 12 weeks. Compared to TC-24 arm, the decrease in TUGT was 0.213 (95% CI −0.382 to −0.044, *p* = 0.013) lower from baseline to 6 weeks and 0.576 (95% CI 0.091 to 1.062, *p* = 0.02) lower from baseline to 12 weeks in the MTC arm (Table 2 and Table 3). The overall change in balance performance and time × arm interaction are illustrated by interaction plots for each index of balance in Figure 3. 

Functional fitness of lower limbs (CST) improved in both Tai Chi arms: compared with baseline, participants were 0.401 (95% CI −0.508 to −0.294, *p* < 0.0005) seconds faster at 6 weeks, and 0.922 (95% CI −1.315 to −0.528, *p* < 0.0005) seconds faster in completing CST. In addition, compared to the TC-24 arm, participants in the MCT arm were 0.221 (95% CI −0.397 to −0.045, *p* = 0.014) seconds faster in completing the CST at 6 weeks, as indicated by a significant time × arm effect observed at 6 weeks (*p* = 0.014). 

### 3.3. Aerobic Exercise Capacity

The 6MWT-measured aerobics exercise capacity improved in the overall study population at 6 weeks, but was not maintained at 12 weeks. Significant time × arm interaction effects were detected at both 6 weeks (*p* = 0.018) and 12 weeks (*p* = 0.003). The MTC arm appeared to have a greater improvement in the aerobics exercise capacity, namely 0.256 minutes (95% CI −0.46 to −0.043, *p* = 0.018) faster in completing the 6MWT comparing to the TC-24 group; this greater improvement was also observed at 12 weeks, with the MTC arm being 0.498 second (95% CI −0.828 to −0.168, *p* = 0.003) faster in completing the 6MWT compared to the TC-24 group (Table 2, visually illustrated in Figure 4).

## 4. Discussion

Previous experimental work demonstrates that TC, when compared to a non-TC control group, is effective in enhancing numerous physiological, psychological, and cognitive outcomes. What is lacking in the literature is a side-to-side comparison between various forms of TC. To address this gap in the literature, the present experiment, among older adults, juxtaposed two forms of TC (TC-24 vs. MTC) on key health parameters that are predictive of premature mortality risk [54,55]. Our main findings were twofold: (1) both forms of TC were effective in enhancing global cognitive function [56,57], balance, and fitness, but (2) MTC was more effective than TC-24 in enhancing these health-related parameters. Given that our previous work has already demonstrated and discussed the beneficial effects of TC on enhancing various health outcomes [12,13,14,15,16,17], the discussion that follows will focus on the unique effects of a modified form of TC (i.e., MTC). 

In the present intervention study, we demonstrated that MTC was superior to TC-24 in enhancing physiological (fitness), neuromuscular (balance), and cognitive outcomes. When compared to TC-24, MTC involves more complex movement patterns [58]. This is important as previous work suggests that more complex movement patterns may be more effective in enhancing neural plasticity [59] and key brain-derived neurotrophins, which play an important role in cognitive function. This is also supported by recent work showing that more complex and coordinated motor martial art exercises, which resemble movement patterns associated with MTC, are effective in enhancing working memory capacity [60,61,62], a cognitive parameter that was tapped with our cognitive assessments. Further, complex movement patterns, when compared to less complex movements, are more effective in increasing regional blood flow and cortical excitability [63,64], which may have important implications in subserving cognitive function and balance performance. These are potential mechanisms that may help elucidate our observed greater effects of MTC (vs. TC-24) on cognitive function and balance. Enhanced cognitive function and balance, in turn, may also have been responsible for the observed improvements in fitness. Our functional fitness parameters (stand-up-and-go, walking task) require adequate strength and balance for optimal performance. Further, as we have demonstrated previously [65], cognitive function is associated with the same aerobic capacity assessment (walking task) employed in our present study. Of course, these interrelationships are complex, as there is likely a bi-directional relationship between cognitive function and aerobic capacity [66]. Nonetheless, our intervention findings suggest that more complex forms of TC, such as MTC, may be optimal in enhancing key health-related parameters (e.g., cognition, balance, aerobic capacity). 

Limitations of this study include not having a non-active control group. However, we intentionally did not employ a control group, as previous work has already thoroughly demonstrated that TC, when compared to a non-active control group, is superior in enhancing various health outcomes, such as those employed in our study. Future work on this topic, however, may benefit from inclusion of a control group, as it will more definitively provide evidence of a causal relationship between different forms of TC on health outcomes. Secondly, the subjects included were all Chinese. The results need to be replicated in other countries before we can conclude that they can be generalized to other populations. Furthermore, while the age of participants ranged from 55 to 79 years, to increase the statistical power to answer our initial research questions, the impact of subgroups classified by age was not further examined in this study. Thirdly, this study did not provide longer term follow-up beyond 12 weeks. Therefore, there is no information on whether the results are sustained over longer periods. Strengths of this study include the study’s novelty, extended intervention period (i.e., up to 12 weeks), and inclusion of multiple health outcomes. Assessors were not blinded to group allocation, so that could be a source of bias. Future studies should pay attention to the following aspects: (1) using functional near infrared spectroscopy to check mechanisms related to global cognition [67]; (2) the possibility to measure Functional near-infrared spectroscopy signals during movement (balance tasks) [68]. 

## 5. Conclusions

In conclusion, our intervention demonstrates that a more complex form of TC (i.e., MTC) is superior in enhancing cognitive function, balance, and aerobic capacity among older Chinese adults. It would be worthwhile for future work to investigate the long-term maintenance effects of our findings. Further, such work should consider evaluating whether different populations, as well as those with varying experiences of TC, moderate the effects of MTC on health outcomes. 

## Figures and Tables

**Table 1:** Summaries of baseline variables in both experimental arms (*n* = 78).

	TC-24 Group	MTC Group
Gender		
Male (*n*)	18	19
Female (*n*)	22	19
Educational level *		
Middle school or below (*n*)	1	19
Associate degree (*n*)	21	13
College or above (*n*)	18	6
Age (years)	59.55 ± 8.91	58.26 ± 7.05
BMI * (kg/m^2^)	24.04 ± 2.66	25.21 ± 2.00
Heart rate (beats/minute)	73.55 ± 8.30	75.53 ± 5.56

Notes: * Variables that turned out to have significant differences of means between the two Tai-Chi arms in our sample and were thus included as main effect terms in all generalized estimating equations (GEE) models to adjust for their confounding effects.

**Table 2:** Parameter estimation and significance of coefficients in the GEE models.

Variable	Time Effect				Group Effect		Interaction Effect			
	Time 2		Time 3		MTC		Time2 × MTC		Time3 × MTC	
	*Coef. (Cohen’s d)*	*p*	*Coef.* *(Cohen’s d)*	*p*	*Coef.* *(Cohen’s d)*	*p*	*Coef.* *(Cohen’s d)*	*p*	*Coef.* *(Cohen’s d)*	*p*
MoCA	1.87 (1.091)	<0.0005	3.449 (1.951)	<0.0005	0.487 (0.769)	0.031				
Left leg balance (eyes closed)	0.754 (0.3)	<0.0005	1.189 (0.558)	<0.0005	0.633 (0.742)	0.396	0.446 (0.457)	0.044	1.485 (1.203)	<0.0005
Right leg balance (eyes closed)	6.199 (0.216)	0.242	1.181 (0.483)	<0.0005	0.057 (0.176)	0.945	−5.065 (0.212)	0.34	1.385 (0.918)	<0.0005
TUGT	−0.370 (0.181)	<0.0005	−0.874 (0.471)	<0.0005	−0.366 (0.48)	0.578	−0.213 (0.558)	0.013	−0.576 (0.523)	0.02
CST	−0.401 (0.248)	<0.0005	−0.922 (0.531)	<0.0005	−0.022 (0.228)	0.971	−0.221 (0.555)	0.014	−0.116 (0.104)	0.642
6-MWT	−0.266 (0.395)	0.002	0.062 (0.188)	0.681	−0.051 (0.483)	0.842	−0.256 (0.529)	0.018	−0.498 (0.661)	0.003

**Table 3:** Mean and standard deviation (M ± SD) of the raw data values and the data values adjusted for confounder effects (the latter are given in parentheses), for cognitive function, balance, and physical fitness indices at baseline, Week 6, and Week 12 in the TC-24 group and MTC group.

Variable	TC-24 Group			MTC Group		
	Baseline	Week 6	Week 12	Baseline	Week 6	Week 12
The Montreal Cognitive Assessment	26.62 ± 1.39 (24.98 ± 1.65)	28.38 ± 1.27 (26.73 ± 1.45)	29.90 ± 0.38 (28.26 ± 1.4)	26.18 ± 1.39 (26.38 ± 1.64)	28.18 ± 1.43 (28.38 ± 1.42)	29.82 ± 0.51 (30.01 ± 1.65)
Left leg balance (eyes closed)	3.86 ± 2.55 (7.24 ± 2.93)	4.61 ± 2.65 (7.99 ± 2.95)	5.04 ± 2.74 (8.43 ± 3.13)	3.58 ± 2.55 (9.04 ± 2.87)	4.78 ± 3.32 (10.24 ± 3.57)	6.25 ± 3.44 (11.71 ± 3.73)
Right leg balance (eyes closed)	3.29 ± 2.23 (8.13 ± 3.87)	9.48 ± 33.56 (14.33 ± 33.3)	4.47 ± 2.05 (9.31 ± 3.61)	3.95 ± 3.32 (6.88 ± 3.9)	5.08 ± 3.59 (8.02 ± 4.14)	6.52 ± 3.33 (9.45 ± 3.97)
TUGT	10.10 ± 2.59 (8.11 ± 2.59)	9.73 ± 2.5 (7.74 ± 2.54)	9.22 ± 1.9 (7.24 ± 2.24)	10.11 ± 2.33 (7.18 ± 2.57)	9.52 ± 2.29 (6.6 ± 2.6)	8.66 ± 1.66 (5.73 ± 2.08)
CST	9.15 ± 2.20 (3.35 ± 2.17)	8.75 ± 2.12 (2.95 ± 2.11)	8.23 ± 1.46 (2.43 ± 1.31)	9.59 ± 2.08 (3.02 ± 2.01)	8.97 ± 1.95 (2.4 ± 1.91)	8.55 ± 1.76 (1.99 ± 1.77)
6-MWT	5.10 ± 1.17 (5.39 ± 1.15)	4.83 ± 1.12 (5.13 ± 1.1)	5.16 ± 0.93 (5.45 ± 0.95)	5.13 ± 0.78 (5.19 ± 0.86)	4.61 ± 0.70 (4.67 ± 0.74)	4.70 ± 0.63 (4.75 ± 0.69)

Notes: TC-24 = 24-style Tai Chi; MTC = Modified Chen-style Tai Chi; MoCA = the Montreal Cognitive Assessment; TUGT = Timed Up and Go Test; CST = Chair Stand Test; 6MWT = Six-Meter Walk Test.
